# International organizations and climate change adaptation: A new dataset for the social scientific study of adaptation, 1990–2017

**DOI:** 10.1371/journal.pone.0257101

**Published:** 2021-09-10

**Authors:** Ece Kural, Lisa Maria Dellmuth, Maria-Therese Gustafsson

**Affiliations:** 1 Department of Economic History and International Relations, Stockholm University, Stockholm, Sweden; 2 Department of Political Science, Stockholm University, Stockholm, Sweden; University of Glasgow, UNITED KINGDOM

## Abstract

This article introduces a new dataset on the climate change adaptation activities of international organizations (IOs). While climate change adaptation has been studied at the local level and in the context of major climate organizations, such as the United Nations Framework Convention on Climate Change, we provide a first quantitative dataset on non-environmental IOs that can be linked to different social scientific datasets relevant for adaptation. Our new dataset contains information on the governance activities of 30 IOs from 1990 to 2017. Based on this dataset, we introduce different types of adaptation-related activities and develop a quantitative measure of IOs’ climate adaptation engagement. We map the adaptation engagement of the 30 IOs across organizations, across issue areas, and over time. This dataset can be used to compare adaptation activities across and within IOs, but also as an empirical foundation for the emerging research field of global adaptation governance, for which IO climate change adaptation activities are relevant.

## Introduction

Long viewed as a local and technical issue, human adaptation to climate change (hereinafter referred to as “adaptation”) was catapulted to the top of the international climate agenda in 2007 when states agreed on the Bali Action Plan under the auspices of the United Nations Framework Convention on Climate Change (UNFCCC). In this plan, an adaptation fund was created, and adaptation was framed as one of the four pillars of climate action, along with mitigation, technology, and financing. In its *Synthesis Report*, the Intergovernmental Panel on Climate Change (IPCC) (2014) confirmed that addressing adaptation challenges effectively depends on policies and cooperation at all levels, including the global level [1]. In the 2015 Paris Agreement, adaptation is recognized as a global goal (Article 7.1) and policy challenge (Article 7.2).

We refer to adaptation as any changes in socio-ecological systems in response to actual or expected climatic stimuli or their effects, which moderates harm or exploits beneficial opportunities [2]. Thus, the notion of adaptation includes different types of activities aimed at reducing exposure and vulnerability to, and improving resilience to, climate change. The need for adaptation stems from insufficient efforts to reduce carbon emissions. The pace of global warming has accelerated so that average global temperature rise is likely to surpass the Paris Agreement goal of 1.5°C between 2030 and 2052 if our emissions continue at their current rate. To be sure, ongoing mitigation efforts such as an increased reliance on renewable energy sources exist, but are overall insufficient, requiring communities and states to find ways to adapt to the diverse sets of risks associated with a changing climate, such as water shortage, food insecurity, and extreme weather events [3]. Adaptation to climate-related risks in areas such as health, development, and security, are becoming ever more pressing.

The increasing global attention to the need to step up adaptation action has paralleled growing engagement by international organizations (IOs) with the issue. A notable trend in global governance is that *non-climate* IOs such as the World Bank (WB) and the World Health Organization (WHO) are increasingly engaging with adaptation. However, engaging with adaptation might crowd out scarce resources that IOs require to pursue their core mandates. And, adaptation funding is scarce: A recent study on climate-related investments (private, public, and multilateral) shows that in 2017–18 the support for adaptation only slightly exceeded 5 percent of total global climate finance [4]. Funding is, thus, no obvious incentive to engage with adaptation. Why are non-climate IOs engaging in issue expansion to address adaptation challenges?

Understanding this puzzle is important given that the actions of IOs matter for local, national, and international adaptation outcomes. In general, IOs adopt policies, provide aid, promote rules and norms, act as policy advisors, contribute to setting the domestic political agenda, and ignite debates with the aim of enhancing public benefit [5–7]. In the area of adaptation, IOs carry out such important roles as well, in particular by funding adaptation-related projects and setting guidelines for local and national adaptation activities [8]. For instance, the United Nations Development Programme (UNDP) has played an important role in supporting national adaptation planning in developing countries [9]. A first step is to better understand how and to what extent IOs engage in adaptation governance.

The social science literature on global climate governance is large and burgeoning. It mainly deals with the topic of climate change mitigation and the UNFCCC [10–12]. Within this literature, an emerging strand of research has begun to foreground adaptation, mapping global adaptation governance activities and evaluating how and when these activities are effective and democratic [13–15]. However, the overwhelming majority of studies on adaptation governance have analyzed local or national adaptation, and not global processes [8, 10], and have mainly relied on case studies of individual political processes, issue areas, or IOs.

Thus, most social science literature on adaptation has been mainly preoccupied with political responses at the local and national level [16–18], for example pertaining to Nationally Determined Contributions submitted to the UNFCCC [19]. Although several IOs have defined the concept of adaptation in relation to their mandates [14, 20], the population of global actors involved in adaptation governance and the degree and determinants of their engagements remain unclear, which hampers the interdisciplinary study of adaptation in the social sciences [8].

An important challenge in this research has been to define and measure adaptation [21]. The ambiguity of the concept as a political problem has made it challenging to evaluate the levels, patterns, and effectiveness of different adaptation responses [22]. As adaptation has been typically seen as a localized issue, the concept and its measurement are even more contested and ambiguous at the global level [23], and definitions of adaptation vary across issue areas. To systematically compare IO engagements with adaptation across issue areas and over time, we conceive of adaptation engagement as a set of activities, including decisions and processes, aimed at reducing the adverse impacts of climate change [24, 25]. In order to qualify as an engagement with adaptation, IOs must explicitly state that an activity is aimed at counteracting the adverse impacts of climate change, but it does not necessarily have to explicitly refer to the notion of adaptation.

Discussing the literature on IOs, it has focused on the structures, activities, and performance of IOs across a large number of issue areas, including climate change. In this large literature, the body of work on issue expansion is particularly relevant. In this tradition, scholars are interested in how, why, and to what extent IOs move beyond their core mandates and become active in new issue areas [5, 6, 26, 27]. Several studies have shown how IOs have expanded their core mandates to address adaptation challenges. The WHO has moved beyond its core mandate in public health to also address climate-related health risks [28]. The International Organization for Migration (IOM) has expanded its core mandate by making climate-induced migration a priority in recent years [14]. The United Nations Security Council (UNSC) has related to climate risks in some of its peace-keeping operations and official statements [29]. The European Union (EU) has been shown to be a frontrunner in linking energy and climate change [30, 31]. However, scholars have yet to provide a large-scale mapping of engagement with adaptation; existing datasets on IOs’ climate governance are typically limited to energy and environmental affairs [32–34]. In the most encompassing assessment of IOs’ policy scope so far, the 29 issue areas covered do not include adaptation [35]. In sum, the absence of suitable data has hampered progress in understanding to what extent, how, why, and with what consequences non-climate IOs engage with adaptation.

We have therefore developed a dataset at the IO-year level including measures of IOs’ engagement with adaptation, which enables us to map adaptation governance across IOs, across issue areas, and over time. We refer to IO engagement as the degree to which IOs govern adaptation in the context of their core mandates by organizing different activities, such as publishing scientific case studies and implementing adaptation programs. The dataset includes several measures as well as a composite index of IOs’ adaptation engagement, thereby enabling systematic assessments of how, to what extent, why, and with what consequences non-climate IOs engage in adaptation governance.

More specifically, the aim of this article is to introduce a novel dataset on the engagement of 30 IOs with adaptation from 1990 to 2017, and map IOs’ adaptation engagement comparatively. To operationalize engagement with adaptation, we introduce different types of adaptation activities and develop a first quantitative measure of adaptation engagement available at the IO level. We use these data to map these organizations’ engagement with adaptation, showcasing how this engagement covaries with climate funding, and discussing the theoretical puzzles that can be addressed using the dataset. Taken together, this dataset can be used to examine issue expansion among IOs in the area of climate change, and to compare, describe, and explain adaptation activities across and within IOs and over time.

## Materials and methods

In this section, we lay out the overarching research design, present the rationale for selecting IOs, issue areas, and the time period, and discuss the data generation process as well as the measurement of IOs’ adaptation engagement.

### Design of the dataset

The design of the dataset was guided by three main choices. The first pertains to the inclusion of a large number of IOs. We selected 30 IOs for which we conducted a qualitative textual analysis of these IOs’ annual reports during the period 1990–2017. As noted, existing research has largely focused on the UNFCCC. Little is known about non-climate IOs, especially the regional ones, such as the Pacific Islands Forum (PIF) or the East African Community (EAC), both of which have engaged with climate adaptation in various ways. We have thus constructed the dataset by including a large number of IOs from multiple issue areas and all world regions. This design choice enables a comparative inquiry and allows us to provide a broad understanding of IOs’ engagement with adaptation.

The second choice concerns the longitudinal character of the dataset. The ways in which IOs have responded to adaptation are mostly studied with a view to the 2010s [14], and a systematic longitudinal analysis is still lacking. We settled for the time period 1990–2017 in order to capture early developments in adaptation engagement, which paralleled the negotiations leading up to the inception of the UNFCCC initiated in 1990. We consider this time period to be sufficiently long to capture long-term developments.

Third, we relied on IO annual reports which provide useful information about IOs’ adaptation engagements. Through annual reports, IOs take stock of their activities in the preceding year. Crucially, the advantage with this data source is that it allows us to compare the content of these texts across different IOs, due to their similar function and structure. Formal rules and other outputs, such as communiqués or guidelines issued by IOs are often not functionally equivalent and therefore not comparable. For instance, UNSC formal statements are not comparable to formal statements by the United Nations High Commissioner for Refugees (UNHCR), as the former reflect the official stance of the Council adopted at a Council meeting and issued as an official document, whereas the latter represent the stance of the Higher Commissioner and not formally adopted decisions.

A known challenge when using annual reports is that these are official documents containing selective and strategic information, in part used to legitimate the IOs’ own actions [36, 37]. Early engagement before 2007 may be underreported, as adaptation has only after the IPCC report in 2007 [2] been recognized as an issue that needs integrating in different issue areas [8]. Therefore, we opted for a broad approach by coding all activities that mentioned activities addressing climate risks as adaptation engagement, and not only those activities that explicitly used the adaptation label. An additional challenge is that documents were not available in all cases (S3 Table in S1 File), which is important to note to interpret average engagement levels analyzed in this article. Taken together, analyzing annual reports enables a systematic comparison of adaptation engagement over time. Next, we discuss the rationale behind the selection of IOs and issue areas.

### Selection of international organizations

The Correlates of War dataset includes 533 IOs [38] whereas the *Yearbook of International Organizations* encompasses as many as 7,722 IOs, which includes non-governmental and hybrid institutions [39]. Here we define IOs on the basis of five criteria. First, IOs are intergovernmental and engage in decision-making, have permanent headquarters, and have a written constitution or construction. Second, an IO has a formal bureaucratic structure (i.e., a legislative body, execution and administration). For instance, the Arctic Council’s secretariat became operational only in 2013, which is why we excluded the organization from the sample. Third, an IO has stable state membership [40] and, fourth, has been active during the period 1990–2017. Fifth, organizations with predecessors, such as The Africa Rice Center, formerly known as the West Africa Rice Development Association (WARDA), are treated as one entity.

The sample of IOs was created in three steps. In a first step, we limited the sample to non-climate IOs. As we are interested in issue expansion, the dataset includes IOs whose founding treaties or legal frameworks do not include climate change. For example, we excluded the UNFCCC whose core mandate encompasses adaptation. The second step consisted of limiting the sample further to IOs in issue areas for which adaptation is relevant. In order to identify the relevant issue areas, we conducted careful desk research by engaging with the case study literature on adaptation [10, 41, 42]. We identified nine broad issue areas: development, disaster risk management, food and agriculture, global development banking, health, migration, peace and security, regional cooperation, and trade. Issue areas such as finance and taxation were excluded. Third, we selected the major IOs in each issue area. For example, in the area of health, we included the United Nations Population Fund (UNFPA) and WHO but excluded the Pan American Health Organization (PAHO). United Nations (UN) specialized agencies and offices were included in the sample if they enjoy autonomy over their operations and budgetary decisions, and have separate membership criteria although some have reporting requirements to the UN General Assembly.

The final sample consists of major IOs with mandates in areas relevant for adaptation, whereby IOs varyingly engage in adaptation, ranging from no engagement to very high levels of engagement. Table 1 shows that this sample of 30 IOs spans all world regions—Africa, Asia, the Americas, Europe, and organizations with global membership—with core mandates in the nine issue areas mentioned earlier. The world region that is best represented is the global level (15 IOs), followed by the African region (6 IOs), the Asian region (4 IOs), the European region (3 IOs), and the North and South American regions (2 IOs).

**Table 1:** Overview of issue areas and IOs included in the dataset.

Policy Field	Organization
Global Development Banking	African Development Bank (AFDB), Asian Development Bank (ADB), European Bank for Reconstruction and Development (EBRD), Inter-American Development Bank (IADB), World Bank Group (WB/IBRD)
Regional Cooperation	African Union (AU), Association of Southeast Asian Nations (ASEAN), East African Community (EAC), European Union (EU), Organization of American States (OAS), Pacific Islands Forum (PIF), South Asian Association for Regional Cooperation (SAARC)
Development	Organization for Economic Cooperation and Development (OECD), Southern African Development Community (SADC), United Nations Development Programme (UNDP), United Nations Children’s Emergency Fund (UNICEF)
Food & Agriculture	Food and Agriculture Organization (FAO), International Fund for Agricultural Development (IFAD), West Africa Rice Development Association (WARDA), World Food Program (WFP)
Migration	International Organization for Migration (IOM), UN High Commissioner for Refugees (UNHCR)
Peace & Security	North Atlantic Treaty Organization (NATO), United Nations Security Council (UNSC), Organization for Security and Defense and Cooperation in Europe (OSCE)
Trade & Economy	Economic Community of West African States (ECOWAS), World Trade Organization (WTO)
Health	United Nations Population Fund (UNFPA), World Health Organization (WHO)
Disaster Risk Management	United Nations Office for Disaster Risk Reduction (UNISDR), United Nations Office for the Coordination of Humanitarian Affairs (UNOCHA)

We do not claim that this sample is strictly representative for the full population of IOs. One should therefore be cautious about generalizing results beyond these IOs and issue areas. However, the crucial advantage of our selection procedure is that it allows us to explore patterns and conditions for adaptation engagement in organizations with varying characteristics, which are often expected to shape issue expansion, such as authority [40]. At the same time, the selected IOs are key governing organizations within their respective policy domains, lending political importance to any inquiry based on this dataset. Sampling IOs randomly on the basis of existing lists of IOs would likely not have led to a sample with these advantages.

### Data collection and processing

In total, we analyzed 697 annual reports produced by 30 IOs for the period 1990–2017. The data was collected in three stages. First, two researchers compiled these annual reports. Second, where annual reports were not available online, IOs were directly contacted to request missing documents. The third stage encompassed the coding of the reports. Initially, the keywords “climate”, “climatic”, “climate risks”, and “adaptation” were used to detect relevant paragraphs within each report. Although IOs reported on both mitigation- and adaptation-related climate activities, we included only activities geared toward adaptation given the research gap outlined above.

During the coding process, two independent coders ran a keyword search for “climate” and “climate change”. Subsequently, explicit mentions of adaptation activities were included in the dataset, which are operationalized as activities directed at the social, security, energy and economic impacts of a changing climate. The result of this process is the coding of 2,146 adaptation activities that fall into seven distinct categories of governance responses generated inductively (Table 2): statements, events, frameworks, funds, operational activities, reports, and institutional change.

**Table 2:** Types of activities included in the dataset.

Types of activities	
Categories	Definition
Statement	Official statements by an IO.
Event	Events, meetings, conferences organized by an IO (e.g., COP side events, conferences, ExCom meetings, roundtable meetings, and forums).
Framework	Frameworks and strategies.
Fund	Financial responses (e.g., the establishment of funds, pooling of funds).
Operational	Operations within an IO (e.g., projects, vocational trainings, and educational programs).
Report	The publication of scientific reports, case studies and activity reports.
Institutional	Institutionalization, e.g., through the formation of working groups, task forces, departments, initiatives, and public-private partnerships.

“Statements” are measures of instances of official communication by IO secretariats, such as the UN Security Council’s presidential statement on effects of climate change and conflict prevention [43]. “Events” refer to any public events, for instance UNHCR’s side events at UNFCCC conferences, and private gatherings, such as Executive Committee meetings. The category “framework” encompasses strategy and framework documents laying out policy or an action plan for how to respond to adaption. The category “funds” captures the creation of funds to adaptation or the pooling of financial resources to address adaptation challenges. “Operational” activities encompass programs, such as the World Bank’s 2017 data collection initiative on building resilience to climate change in six countries [44], and projects, such as the European Commission’s projects on energy infrastructure [45]. The category “reports” refers to IOs’ publishing results of scientific enquiries, country case studies for instance WHO’s climate impacts on health. The final category captures “institutional” change, including IOs’ structural responses to adaptation. Examples are the working group on the management and emergency response to climatic disasters established by the Association of Southeast Asian Nations (ASEAN) [46] and the Natural Capital Financing Facility set up by the EU to mobilize to generate new private investment in nature and climate adaptation [45].

The coders decided if the text addressing adaptation included an IO’s governance response in one of these categories. We conducted extensive validity and reliability tests. We tested the validity of the coding scheme by applying it to various documents published in different years (1997–2017), and across IOs in pilot coding studies. In addition, we have sought to ensure reliability by giving two coders the task of conducting the document analysis independently from each other. In total, 35 percent of the documents were coded by both coders, and we arrived at a satisfactory level of intercoder reliability for the different items. The intercoder reliability for each category in terms of Krippendorff’s alpha is as follows: statements (0.95), events (0.81), frameworks (0.83), funds (0.84), operational activities (0.91), reports (0.87), and institutional change (0.79). These values indicate substantial agreement between the coders.

The annual reports were subsequently analyzed through qualitative textual analysis. We opted for a qualitative analysis as IOs’ engagement with climate adaptation is still poorly understood [8]. Despite a burgeoning comparative literature on climate adaptation at the national level [47, 48], the notion of adaptation is rather vague and defined differently in different contexts, as elaborated earlier [24]. We therefore needed to gain an in-depth understanding of the ways in which IOs communicate about adaptation in the annual reports before we were able to quantify the information. Alternative approaches, such as supervised and unsupervised machine learning techniques, which are increasingly common in adaptation [49] and international relations research [50], would have performed worse. Any automated text analysis might have glossed over valuable detailed information about IO activities, for instance the degree to which adaptation challenges were prioritized in a specific project. Thus, although the qualitative analysis of 697 official documents was time consuming, it provided much needed in-depth insight into IOs’ activities.

Where the annual reports lacked sufficient information for a particular activity, additional documents were consulted, including project descriptions, briefings, treaty provisions, and policy guidelines. We use this additional material only when a particular adaptation activity is included in the annual report but is insufficiently elaborated upon. As the additional material was used only to complement the existing information in annual reports, this practice does not compromise the comparability of the IO annual reports.

The outcome is a unique dataset of seven categories of adaptation responses of 30 IOs for each year during the period 1990–2017. Next, we discuss how we created the index of IO engagement with adaptation on the basis of these data.

### Measuring adaptation engagement

Previous research provides useful insights for how to measure adaptation at the national level [17, 51, 52]. At the global level, Hall (2016) focuses on policy, rhetorical, structural and operational change to study mandate expansion to integrate adaptation [53]. Persson (2019) emphasizes the importance of the scale of the adaptation problem, level of governance, and actors involved, when assessing global adaptation governance [8]. Taken together, these studies are evidence that IO engagement is a complex and multi-dimensional concept. We therefore propose a measure that takes into account this complexity while at the same time lending itself to making inference to the overall degree of adaptation engagement of an IO.

This measure is a composite index with four dimensions or variables coded for each adaptation activity: prioritization, time horizon, funding, and staffing. These dimensions are mentioned as central aspects of organizational ambition and commitment in the literatures on mainstreaming [54], policy integration [55], interaction management [56], and IOs’ own assessments of their commitments to climate adaptation [57].

Table 3 illustrates how each of the four dimensions were coded by using the example of a specific adaptation activity. The first dimension, prioritization, captures the relative weight that IOs put on adaptation objectives compared to other sectoral goals in a specific reported activity [58] and S1 Table in S1 File). An activity was assigned the value of “primary priority” (= 1) if adaptation is the main focus of that activity. Secondary priority (= 0.5) means that adaptation is a subsidiary topic in an activity. For instance, if a specific report features climate change impacts on human health as the main topic, it is coded as “primary priority” on the prioritization dimension. By contrast, a specific mitigation project including a minor adaptation component is an example of a “secondary priority”. By providing information on the extent or depth of adaptation engagement, the prioritization dimension in part address the issue that an IO may merely relabel an existing activity as adaptation but without actually addressing it.

**Table 3:** Coding example for a specific adaptation activity (program) by the EU in the category “operational”.

Dimensions	Prioritization	Time horizon	Funding	Staffing
**Excerpt from Annual Report**	“. . .focuses on priority area: adaptation …”	“… between 2014 and 2020…”	“…and will provide €864 million …”	“… appointed staff responsible for climate action”
**Coding**	Primary Priority	Long-Term	Yes	Yes
**Weighting Score** ^**a**^	1	1.5	1	1

*Note*: This table includes the variables coded by the author from the IOs’ annual reports included in the IO engagement dataset described above.

^*a*^ The final index score was calculated by first adding the scores, as exemplified in the table, and then by averaging all responses in one year per IO. For further details on operationalization, scoring, and coding, see S1 File.

The second dimension, time horizon, captures the temporal length of an adaptation activity. The variable is coded 0.5 for one-shot engagements with adaptation, 1 for medium-term responses (up to 5 years), and 1.5 for responses lasting longer than 5 years. Longer-term activities are interpreted as greater commitment to climate adaptation (S2 Table in S1 File).

The last two dimensions, funding and staffing, indicate how willing IOs are to use their limited resources on adaptation-related programming. Allocating funding and staffing are commonly referred to in institutional briefings as activities that demonstrate organizational commitment to climate change mitigation and adaptation [59]. The amount of funds and staff allocated to climate adaptation is partly a function of an IO’s total resources, but fund allocations also indicate how willing an IO is to engage with the issue. The funding variable captures whether IOs create or pool funds for an activity (= 1, 0 if otherwise). Staffing refers to whether an IO recruits or assigns staff to an adaptation-related activity (= 1, 0 if otherwise). Thus, both funding and staffing are binary measures, taking a value of 1 if fund creation or staffing is present and 0 if not (see S1 File for more details).

Each of these adaptation-related activities were weighted–or multiplied–by the scores for these four dimensions (weighting scores). Then the activities were summed up for each IO in each year, which yields a specific score for each IO in each year.

Based on these scores for each IO in each year, we calculate the IO Engagement Index in four steps. First, we normalized each variable *x* in the index to bring the values in each variable to the same range (prioritization, time horizon, funding and staffing). This normalization takes the generic form:

$$let\begin{array}{c} ∼\\ x_{it} \end{array}=\frac{x_{it}}{max(x_{it})}$$ (1)

for every *i* and *t*, where *i* refers to an IO and *t* to a year in the dataset. After normalizing each of the four variables on the basis of this formula, we created the additive index by using the normalized variables. The formula for this second step is as follows:

$${\begin{array}{c} ∼\\ {index}_{it}=prioritization \end{array}}_{it}+{\begin{array}{c} ∼\\ timehorizon \end{array}}_{it}+{\begin{array}{c} ∼\\ funding \end{array}}_{it}+{\begin{array}{c} ∼\\ staffing \end{array}}_{it}$$ (2)

In a third step, we normalized the overarching index by dividing the index value of an IO in a given year by the maximum index value across all years.


$${\begin{array}{c} ∼\\ index \end{array}}_{it}=\frac{{index}_{it}}{{max}_{}({index}_{it})}$$ (3)

In a fourth and final step, all index scores were multiplied by 100, resulting in an index ranging from 0 (no engagement) to 100 (maximum engagement).

This index correlates highly with a simple additive index of the four components (prioritization, time horizon, funding and staffing) (*r =* 0.89). By contrast, the IO Engagement Index is adjusted to have a standard minimum and maximum value. The internal reliability of the index dimensions has a Cronbach’s alpha of 0.79, which indicates that the four dimensions consistently measure the IO adaptation engagement.

To explore the validity of the index, we explored the possibility that IOs with larger budgets that might engage more frequently in funding-related and potentially other activities might also score higher on the index. To this end, we examined the relationship between IO annual revenues and IOs’ adaptation engagement, but find little evidence for this. The correlation between IO annual revenue in million United States dollar (USD), derived from the finance and budget statistics of the UN System Chief Executives Board for Coordination [60], and the Adaptation Engagement Index is quite weak (*r* = 0.2527; N = 455), which we illustrate in S1 Fig in S1 File. To illustrate, in 2017 the International Fund for Agricultural Development (IFAD) and UNHCR scored similarly on the index (22.37 and 21.87, respectively) but had very different revenue levels (418.85 and 4226.52 million USD, respectively). To completely rule out a potential bias toward larger organizations on the Adaptation Engagement Index, future studies could expand the dataset to include all IO activities, which would allow for examining the proportion of the total number of adaptation activities to the total number of IO activities. Taken together, our evidence does not suggest that the Adaptation Engagement Index is biased toward IOs with larger budgets.

### Measuring framings of adaptation

In addition to measuring adaptation engagement, we abductively identified four ways in which IOs frame adaptation. Existing framings refer to the ways in which IOs describe the negative impacts of the climate change and who is subject to these impacts. In short, these framings capture how IOs describe adaptation problems.

We identified different framings through a qualitative content analysis of how adaptation activities were phrased in IO annual reports. The four framing types include (a) economic framing (b) human security framing, (c) state security framing and (d) energy security framing of climate adaptation. Economic framings refer to the economic implications of climate change, for example on consumption, production or trade. Human security framings often refer to vulnerability or IOs’ humanitarian activities to alleviate the adverse impacts of climate change on humans, for example in food security programmes. State security framings of adaptation imply that IOs refer to climate impacts as threats to national security, for instance small islands states whose security is threatened by rising sea levels. Lastly, energy security framings of adaptation occur when IOs focus on the disruptions in energy supply chains due to climate change.

## Results

During the period 1990–2017, most of the 2,146 adaptation-related activities of IOs were operational—e.g., on-the-ground projects and country operations (37.27% of all adaptation-related actions identified, *N* = 800 of *N =* 2,146). The second most frequent category is events, such as conferences and workshops organized by IOs (22.50%, *N* = 483). The publication of reports amounts to 13.09% (*N* = 281) of adaptation activities, followed by the creation of funds 11.88% (*N* = 255) and frameworks 10.76% (*N* = 231). Institutional change, for example brought about by establishing departments tasked with adaptation, and setting up task forces as well as working groups, amount to 3.22% (*N* = 69). Specific declarations and statements merely make up 1.26% (*N* = 27) of all adaptation-related actions.

**Fig 1:**
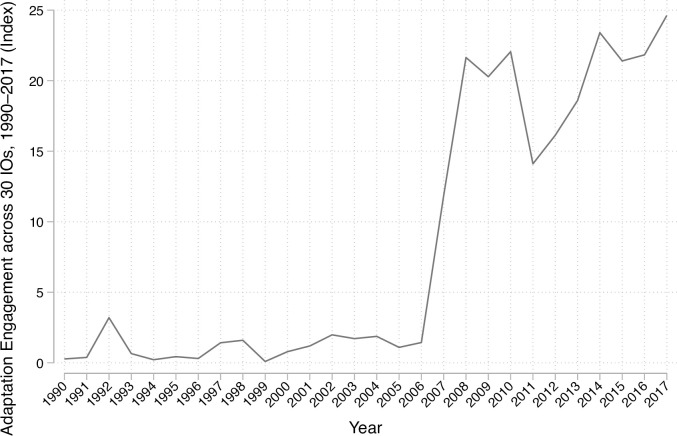
IO Engagement Index across 30 IOs, yearly averages.

The dataset demonstrates that IOs have increasingly engaged with climate adaptation in recent decades. Fig 1 displays the composite IO Engagement Index over time. There is a strong increase in the adaptation engagement of IOs over the 27-year period, evidenced in an increase in the average score of the index from 0.27 out of 100 in 1990 to 24.63 in 2017. The most notable pattern is the steep increase in 2007 and 2008. While the average engagement was 1.37 in 2006, it increased to 11.85 in 2007, and to 20.84 in 2008, displaying a structural break during this time span, after which IO engagement with adaptation has fluctuated.

Low levels of IO engagement with adaptation from 1990 to 2007 can be attributed to the politics of climate change during the 1990s, where global policy focused mostly on mitigation. This changed significantly especially in 2007 when the fourth IPCC report was released and the Bali Action Plan was adopted, which outlined *both* adaptation and mitigation as important pillars of the UNFCCC’s mission [61]. To be sure, some IOs had addressed adaptation even before 2007. The WHO’s study on the climate impacts on health in 1990 is a case in point [62]. However, this was a relatively marginal topic in a specific unit in the organization at the time, and no information was included in the annual reports [13].

**Fig 2:**
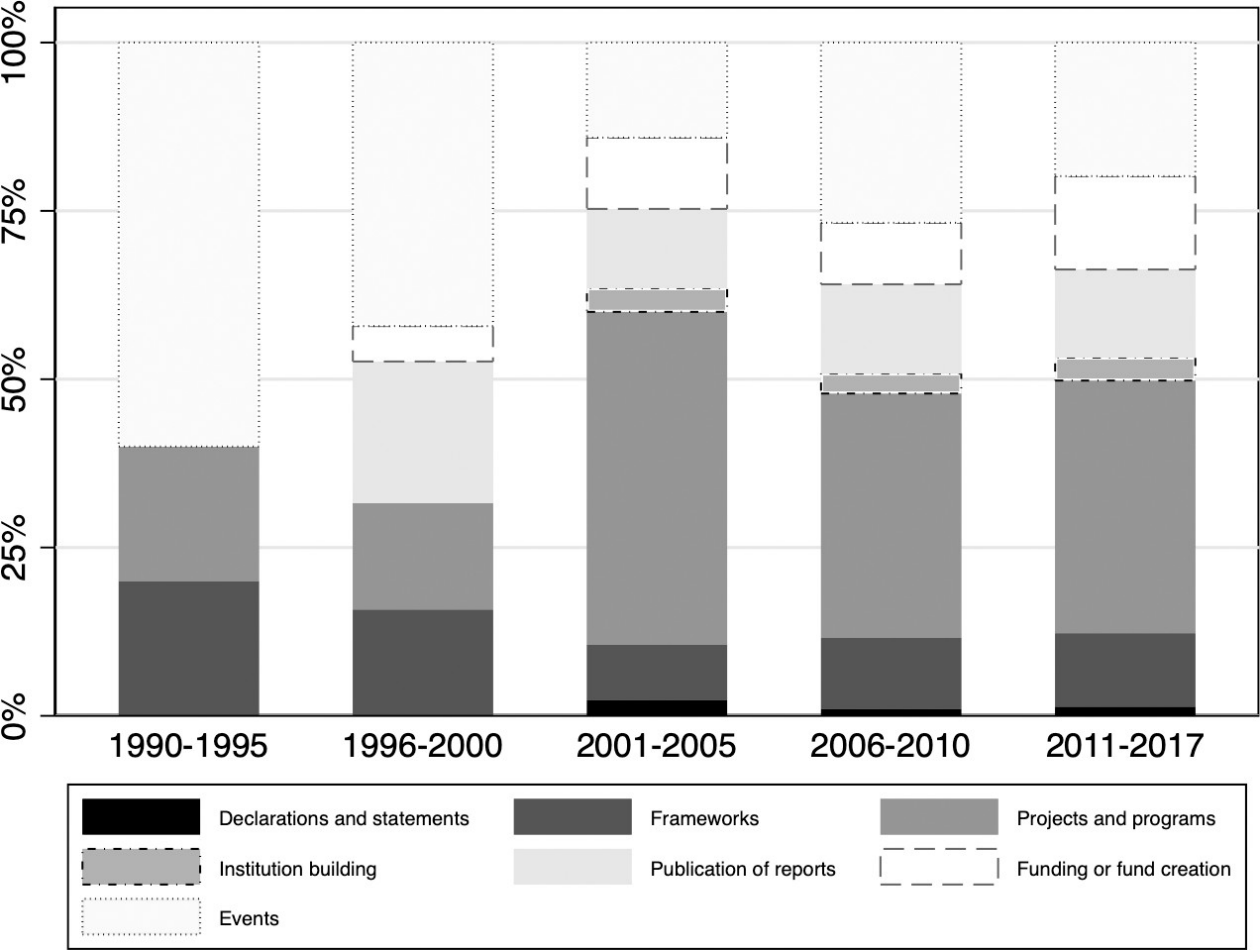
Proportion of the number of IO adaptation activities in seven categories to the total number of adaptation activities in a five-year period, 30 IOs, 1990–2017. Notes: Five-year averages, with the exception of the final period which is a six-year average. Source: IO Engagement Dataset.

This pattern of IO engagement begs important questions about how the shares of specific adaptation activities have developed over time (Fig 2). The 1990–1995 period is characterized by a relatively high share of events and conferences in proportion to all activities. After 2001, local projects and programs began to constitute the majority of how IOs have engaged with adaptation, e.g., the World Bank’s Agricultural Adaptation program for agricultural development and adaptation to climate change [63]. Fund creation activities were mainly established during the period 1996–2000 and proliferated after 2001. A typical example of fund creation is the ASEAN-India Green Fund established in 2010 to support pilot projects of adaptation to and mitigation of climate change [64]. The publication of scientific reports followed a similar trajectory. WHO’s continuous reporting on the health impacts of climate change features prominently in that category. In the final period 2011–2017, we see comparatively little change in the shares of adaptation activities when compared to the preceding time period.

**Fig 3:**
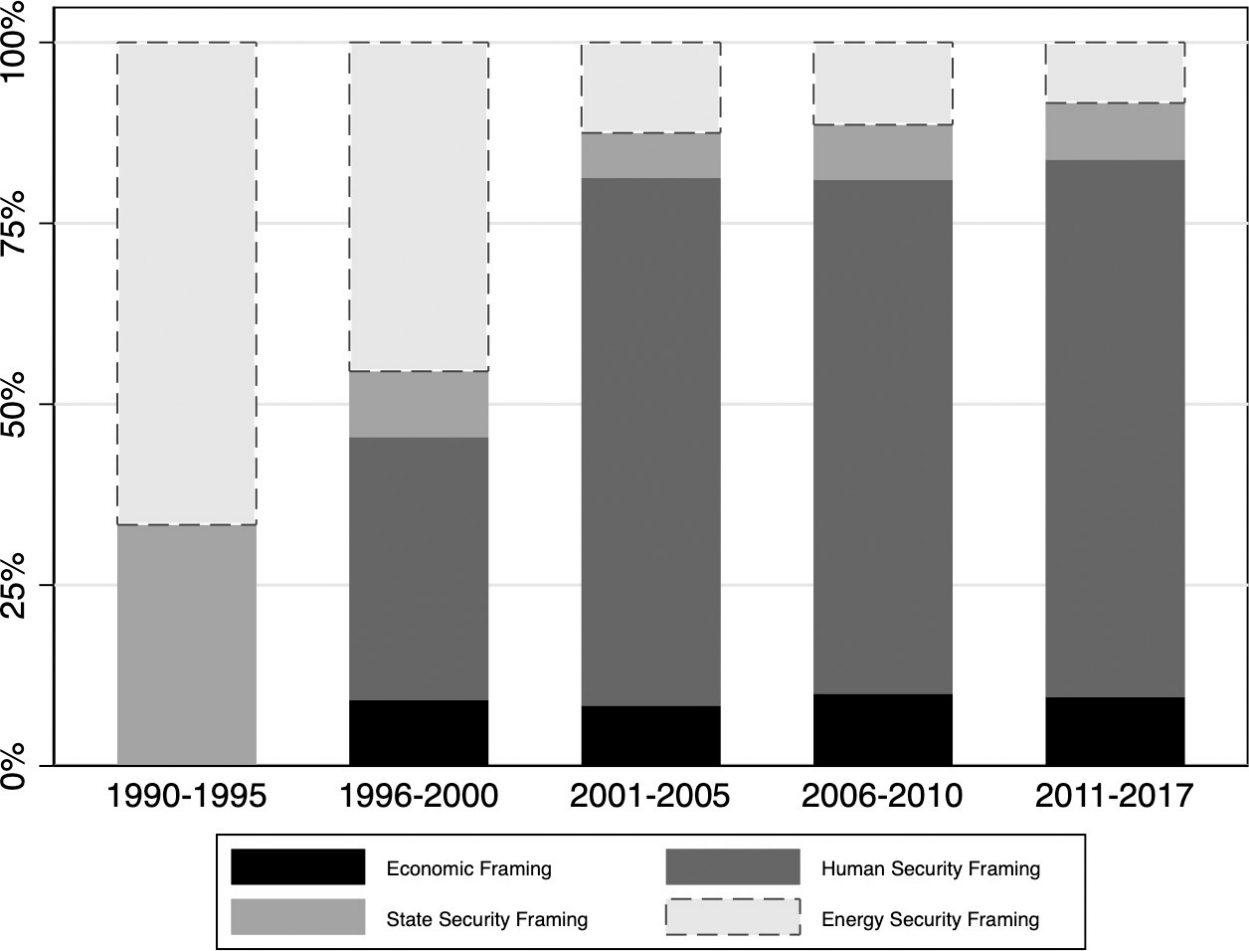
Proportion of the instances of adaptation framings in four categories to the total number of framings in a five-year period, 30 IOs, 1990–2017.

IOs have defined adaptation-related challenges differently over time (Fig 3). As set forth above, there are four main framings of climate adaptation that are often linked to the rationale as to why an IO responded to climate risks. Between 1990 and 1995, energy and state security framings of climate adaptation were most common. Examples are the European Commission’s adoption of a Green Paper on the climate change’s effects on the security of energy supply [65]. After 1996, framings have diversified. To illustrate, the African Union promoted several framings of climate risks, focusing on the potential repercussions of climate change on the global economy and on human security.

After 2001 a human security framing of climate risks became predominant. Illustrations of this type of framing are AU’s and IOM’s attempts to put the link between migration and climate change on the global agenda by highlighting the human security risks [66]. Even IOs that do normally not place climate risks high on their agenda, such as, for instance, trade organizations, adopted a human security framing during this period. This framing was, for instance, clearly adopted in a roundtable organized by the World Trade Organization (WTO) that focused on food security [67].

**Fig 4:**
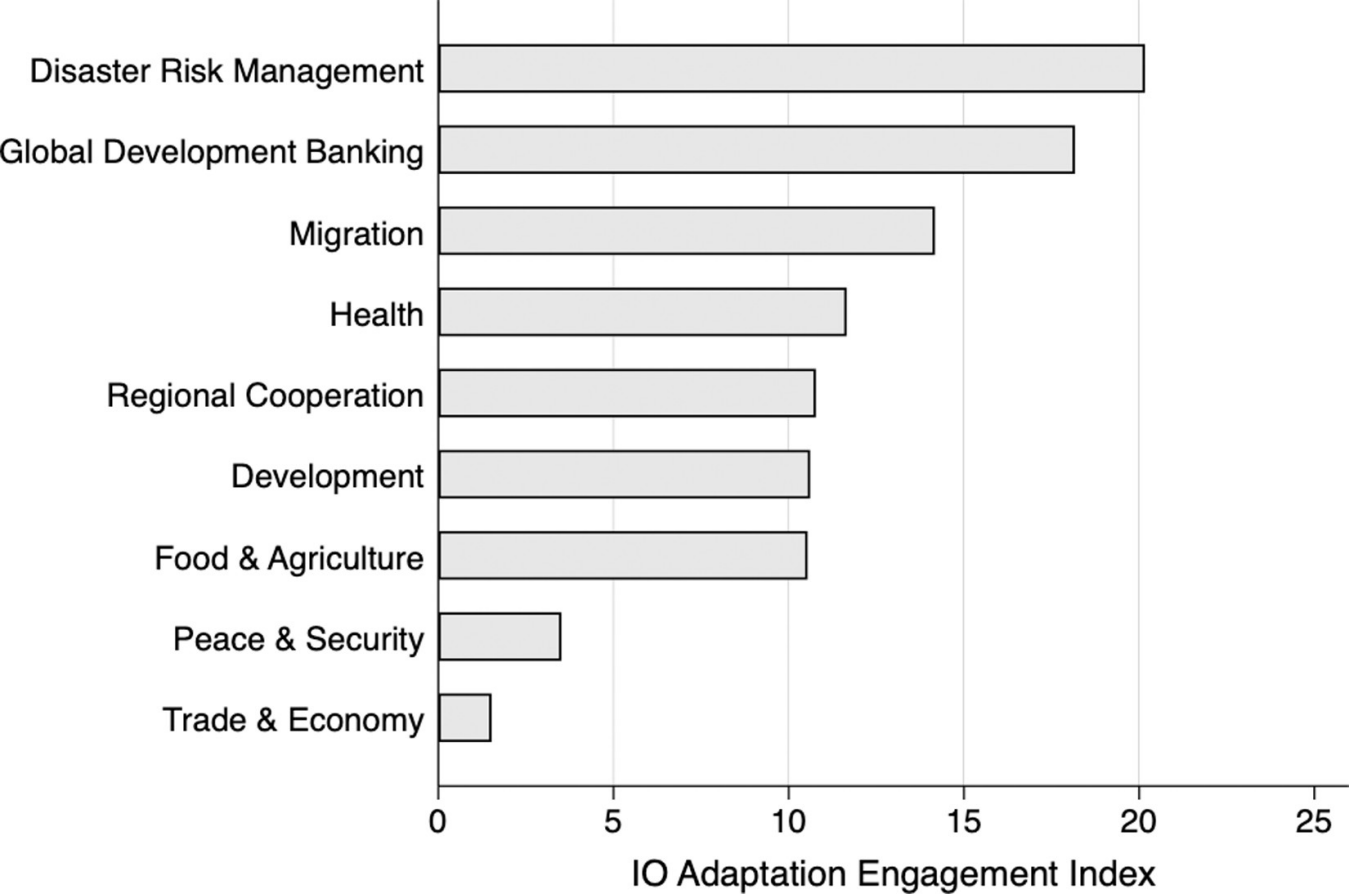
Average IO Engagement Index, by issue area (1990–2017). Notes: This pattern is consistent in most years across the time period observed.

Previous comparative case studies in global climate governance have shown that IO behavior in addressing new problems varies across issue areas [11, 68]. Our data corroborates this assessment. Fig 4 shows the patterns of IO engagement across 9 issue areas, where the index scores represent the mean of adaptation engagement in each issue area. These average scores for the period 1990–2017 suggest that IOs in disaster risk management have most frequently engaged with adaptation, followed by global development banks (Asian Development Bank (ADB), European Bank for Reconstruction and Development (EBRD), Inter-American Development Bank (IADB), World Bank), migration (UNHCR, IOM), health (WHO and UNFPA) and the regional cooperation IOs (e.g., EU, ASEAN, AU). The lowest levels of engagement were found in peace and security (UN Security Council, North Atlantic Treaty Organisation (NATO) and Organization for Security and Co-operation in Europe (OSCE)) as well as trade (WTO).

**Fig 5:**
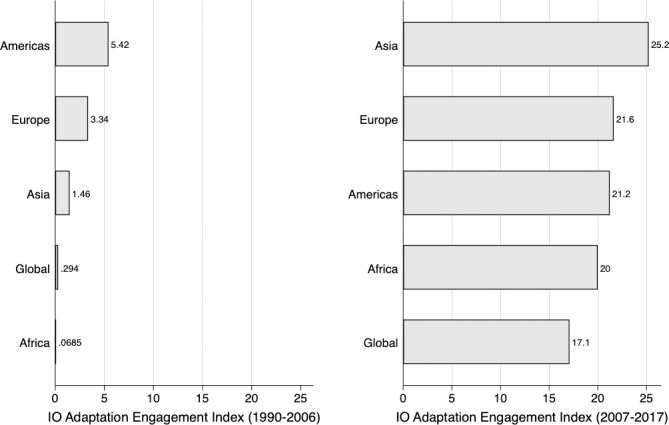
Average IO Engagement Index, by world region (1990–2017).

Engagement with adaptation also varies across IOs when we categorize the sample based on the geographical region (Fig 5). There is great variation before and after 2007, which is why we present the data divided into these two time periods. While IOs in the Americas (IADB and Organization of American States) were the early starters in adaptation governance in the 1990–2006 period, IOs in Asia and Europe were most engaged during 2007–2017 (Fig 5).

**Fig 6:**
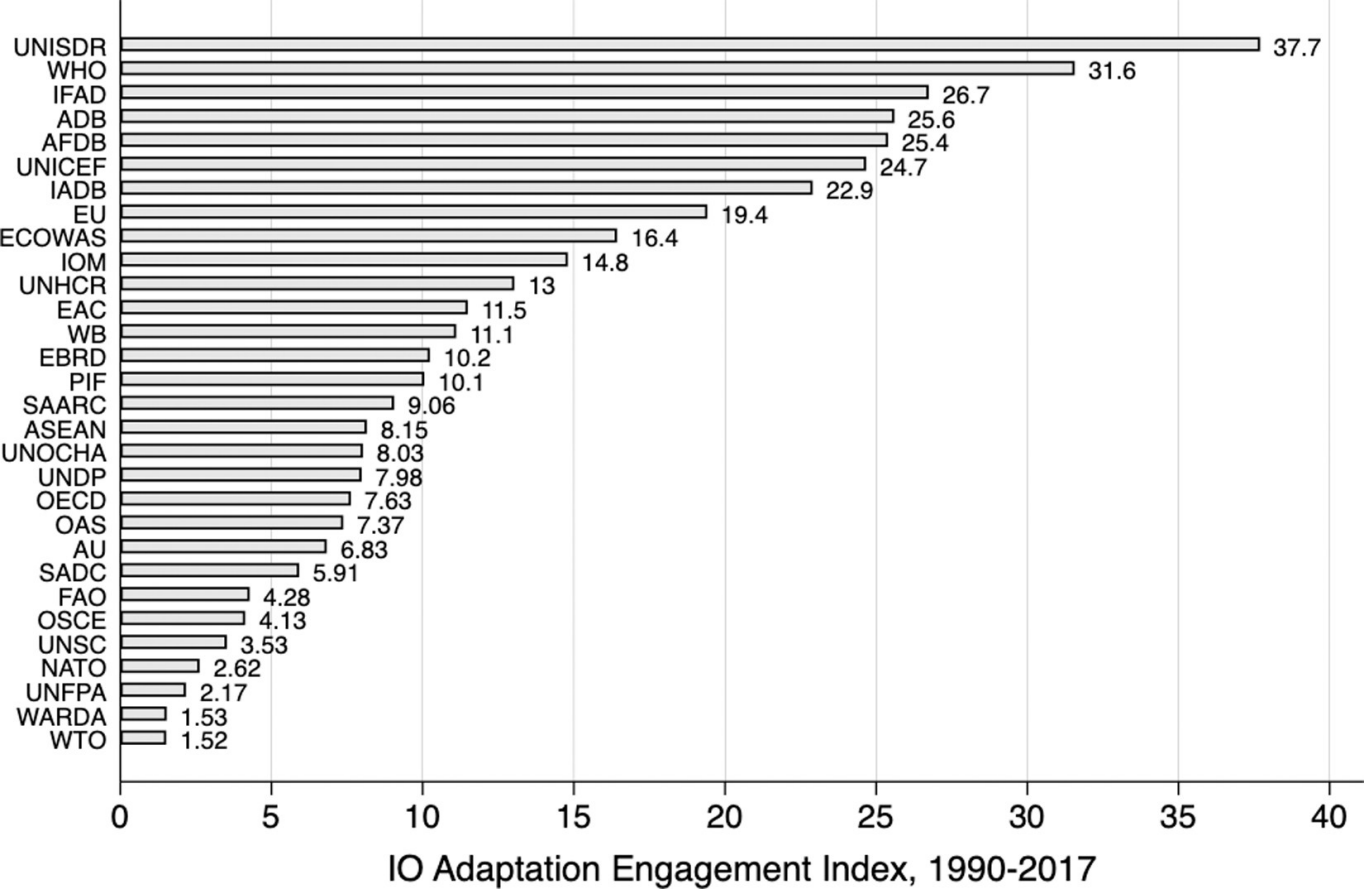
Ranking of IOs, mean of IO engagement index, 1990–2017.

Next, we examine the differences in adaptation engagement across individual IOs. Fig 6 ranks IOs by depicting their average scores in the IO Engagement Index for the 1990–2017 period. UNISDR tops the list, scoring 37.7 out of 100 on the index, followed by WHO, IFAD, ADB, African Development Bank, United Nations Children’s Emergency Fund (UNICEF), IADB and EU, which all have a history of high adaptation engagement. The EU has most often engaged in adaptation in every year until 2001, and in 2014 the EU has the highest engagement score, 100 out of 100. Specialized IOs, such as UNICEF and IOM, have engaged with adaptation, along with IOs with a broader range of issue areas, such as the World Bank and EAC. WTO and the peace and security IOs, such as NATO, OSCE and UNSC, score lowest in comparison to other IOs.

Fig 6 showcases how our comparative and broad selection of non-climate IOs enabled nuanced conclusions about IOs’ adaptation engagement. In fact, some of the most engaged IOs–WHO, UNISDR, IFAD, ADB–have attracted minimal scholarly attention from global adaptation governance scholars. This ranking suggests that focusing only on the climate IOs or on the European organizations would lead to biased conclusions and miss important variation in IO adaptation engagement.

In sum, our analysis has revealed patterns of IO adaptation engagement that beg important questions about their drivers and consequences. Our dataset could be fruitfully matched to other existing datasets at the IO-year level and used to select cases for the purpose of historical or comparative inquiries of specific IOs or political processes.

## Conclusion

In this article, we have presented a novel dataset and mapped the yearly engagement of 30 IOs with climate change adaptation during the period 1990–2017. We wish to highlight two key insights. First, the evidence in this dataset suggests that IOs in nine different non-climate issue areas have expanded their mandates over past decades, increasingly engaging with adaptation. This is a development that spans all observed issue areas and world regions. Second, there is considerable variation in adaptation engagement across IOs, across issue areas, across geographical regions, and over time. Importantly, IOs in disaster risk management and development banking are most engaged, and IOs in trade and security least. European organizations and IOs in the Americas are most engaged, and global as well as African organizations least. Regarding the variation over time, we have seen a surge in adaptation engagement in 2007 after the 2007 IPCC report on adaptation and the Bali Action Plan.

There are three broader implications for the social scientific study of adaptation. To begin with, our evidence is in line with previous research suggesting that adaptation is integrated in different issue areas [13, 14, 29], but provides a more nuanced picture of the patterns of this variation. Starting from this assessment, future research could usefully analyze the patterns, causes, and consequences of IO-specific engagement with adaptation across different sectors of society, in conjunction with increasing cross-border flows and risks. Future work could also extend the analysis of international adaptation engagement to other types of organizations such as companies, partnerships, and civil society organizations, using the insights of our qualitative text analysis as a basis for developing automated text analysis approaches.

Moreover, the evidence provided here can be used for selecting in-depth studies of individual IOs or specific adaptation-related processes and situate those in broader patterns of IO adaptation engagement. Our analysis yields puzzles that may be usefully addressed in future comparative research on adaptation, which is still scant [69]. For example, why do the ASEAN and ADB engage varyingly with adaptation, although they are located in the same region and their members face similar adaptation challenges? ADB is one of the early adapters and ASEAN an organization engaging relatively late with adaptation–why? Our data thus enables future qualitative comparative case studies of specific IOs over time, or comparative case studies of IOs within or across issue areas, seeking to explain variation in engagement.

Finally, the dataset could be used to link to existing large-*n* data on IOs. This could include other aid data channeled through IOs, such as multilateral development or disaster aid, for example available through the OECD’s DAC database, to ascertain if adaptation engagement is supported by or correlated with specific aid flows [70]. Other examples include datasets on IO authority to examine whether IO authority may be a driver of IO adaptation engagement [71]. Yet other studies could usefully link IOs’ varying engagement with adaptation to IO members’ vulnerability and adaptive capacity, for example by using vulnerability indices such as the Notre Dame Global Adaptation Initiative (ND-GAIN) or hazard severity measures available in the Emergency Events Database (EM-DAT) and from meteorological re-analyses [72]. Such extended datasets would enable the comparative study of the causes and consequences of adaptation engagement by IOs across countries, across issue areas, and over time. These issues will become increasingly important to understand as climate-related risks for humans and states are becoming more pressing.

## Supporting information

S1 File (DOCX)
