# Supplementary material for: International organizations and climate change adaptation: A new dataset for the social scientific study of adaptation, 1990–2017
Source: PLoS One. 2021 Sep 10;16(9):e0257101. doi: 10.1371/journal.pone.0257101 (PMC8432833; doi:10.1371/journal.pone.0257101)
Supplement: S1 File — (DOCX) [file pone.0257101.s001.docx]

**Climate Change Adaptation and International Organizations: A New Dataset for the Social Scientific Study of Adaptation, 1990–2017**

**S1 File**

To measure international organizations’ (IOs) engagement with climate change adaptation, the Adaptation Engagement Index is based on a coding of the activities and behavior of IOs. The index is based on information from 30 IOs. The unit of analysis is an IO in a particular year (see Table A3). The data was coded on the basis of annual reports of these IOs for the period 1990-2017. Supplementary formal IO documents, such as strategy documents and activity overviews, were analyzed in the case of missing information in the IO annual reports. All documents are publicly available. The manuscript discusses the sample selection and the different steps undertaken to collect the material in depth.

**Measuring IO Adaptation Engagement**

The notion of “engagement” refers to an IO’s involvement in governance in an issue area outside its core mandate – here, climate change adaptation. The concept comprises a range of organizational activities, including publishing reports, forming advisory groups, and developing new projects that establish or maintain links between an IO’s mandate (e.g., health) and a target (e.g., climate change). Engagement is operationalized as an index on the basis of prioritization, time horizon, funding, and staffing.

1. **Measuring Prioritization**

A core component of IO engagement is the prioritization of climate change adaptation within an activity. An activity is weighted or multiplied by the score of 0.5 if climate adaptation is targeted but is accorded secondary priority to the original area of the activity. A weighting score of 0.5 indicates that the aim of the IO is to avoid contradictory sectoral policies (addressing climate adaptation cross-sectorally in the interest of establishing a coherent policy) or to compensate for adverse climate effects ensuing from their sectoral responses. The secondary priority is closely related to the concept of “coordination” in the mainstreaming literature (1, 2). A weighting score of 1 on the prioritization variable indicates either that a climate adaptation is the main priority of a response or that it is accorded equal priority with another target. Hence, the prioritization variable or dimension reflects the degree to which IOs incorporate climate adaptation in their activities, and is thus an important aspect of IO engagement. See Table A1.

**b) Measuring Time Horizon**

The time horizon variable or dimension measures the planned duration of an activity. Is the activity one that will occur at a single point in time, one that will be extended, or something in between? In the case of a one-shot response, such as publication of a report, the activity is multiplied by the score of 0.5; medium-term activities, such as programs lasting for a couple of years, are multiplied by the score of 1; finally, long-term activities, defined as those lasting longer than 5 years, are multiplied by the score of 1.5. See Table A2.

**c) Measuring Funding**

The funding variable captures whether IOs create funds or pool financial resources to address adaptation challenges. It includes funding used for a specific response, such as a project or program. This binary variable is multiplied by the score of 1 if funds are created or resources pooled, and 0 if otherwise.

**d) Measuring Staffing**

Staffing refers to an IO’s recruitment or assignment of staff for the realization of a response. This binary variable is multiplied by the score of 1 if the staff was assigned to work on the activity, and 0 if otherwise.

**Table S1.** Measuring Prioritization

| **Categories** | **Definition** | **Weighting Score** | **Illustrative examples** | **Source** |
| --- | --- | --- | --- | --- |
| Secondary Priority | Adaptation is given  secondary priority when compared to other goals in a response. | 0.5 | “Unleashing agricultural productivity  in Africa has the potential to ensure  food security and provide gainful  employment for the entire continent…  In addition, the Bank promotes climate-smart agriculture to help build climate resilience.” | World Bank  *Annual Report*,  2017 |
| Primary Priority | Prioritization is primary  priority when compared to  other goals in a response. | 1 | “A joint STDF/World Bank expert  seminar on ‘Climate Change and  Agriculture Trade: Risks and Responses’, …raised awareness among donors and developing countries of the importance of integrating the climate change dimension  into their SPS-related technical assistance programmes. | WTO  *Annual Report*,  2010 |

**Table S2.** Measuring Time Horizon

| **Categories** | **Definition** | **Weighting Score** | **Illustration** | **Source** |
| --- | --- | --- | --- | --- |
| One-Shot Responses | A short-term  response  occurring once  within  an IO. | 0.5 | “On 20 July 2011, the Council held an open thematic debate on the impact of climate change on the maintenance of international peace and security.” | UNSC, Report of the Security Council. 1 August 2010-31  July 2011. General Assembly Official Records. Sixty-sixth Session Supplement No. 22011 |
| Medium-  Term  Responses | A response lasting  up to five years. | 1 | The ASEAN Committee on Science and Technology (COST)’s ASEAN Plan of  Action on S&T (APAST) 2007-2011 has also been expanded  to include health and climate  change as two additional  flagship programmes.” | ASEAN Annual Report 2009 |
| Long-Term Responses | Long-term responses  lasting more than  five years. | 1.5 | “The sixth Community environment action programme was adopted by ... [t]his 10-year programme[.]” | EU Annual Report 2002 |

**Table S3.** Population of International Organizations

| **No.** | **Organization Name** | **Abbr.** | **General Purpose IO** | **IO has GEF Accreditation** | **Regional membership** | **Policy Field** | **Years for which annual reports and documents are available** |
| --- | --- | --- | --- | --- | --- | --- | --- |
| 1 | African Development Bank | AFDB | 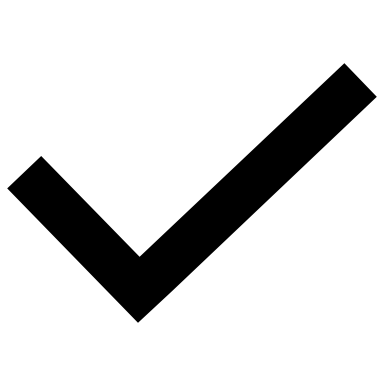 | 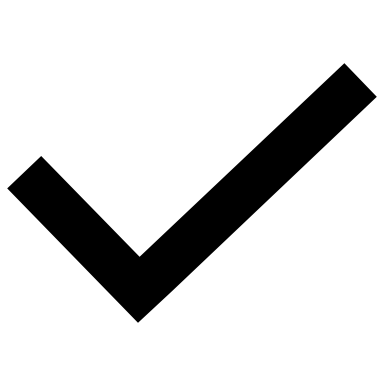 | 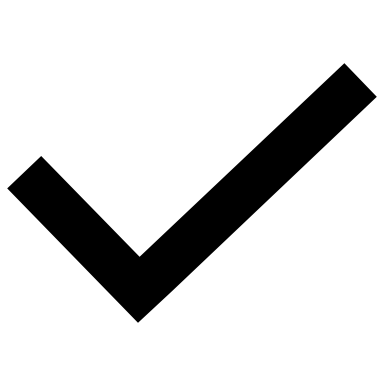 | Global Development Banking | 1997-2017 |
| 2 | African Union | AU | 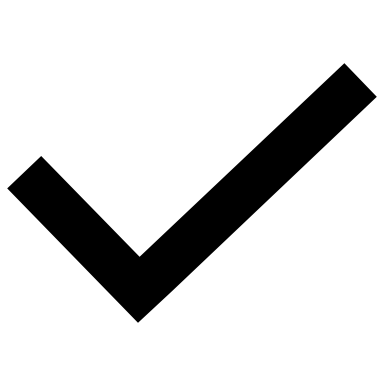 |  | 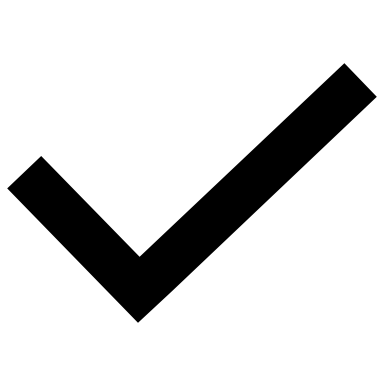 | Regional Cooperation | 1990-2016 |
| 3 | Asian Development Bank | ADB | 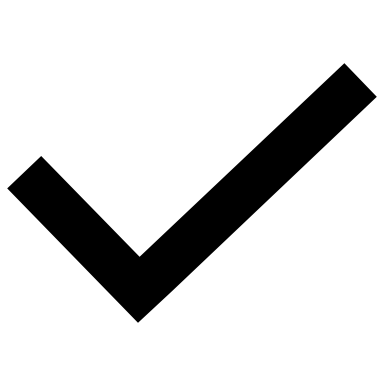 | 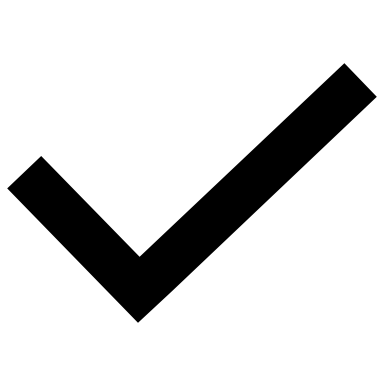 | 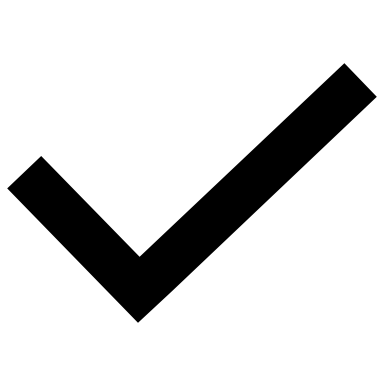 | Global Development Banking | 2000-2017 |
| 4 | Association of Southeast Asian Nations | ASEAN | 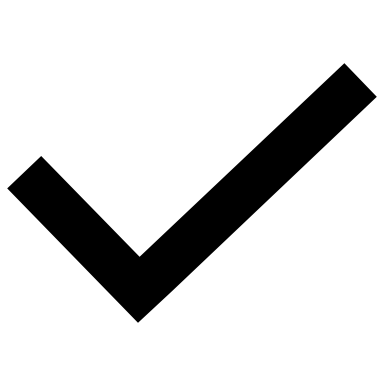 |  | 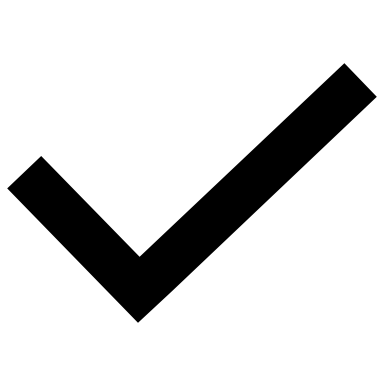 | Regional Cooperation | 1990-2017 |
| 5 | East African Community | EAC | 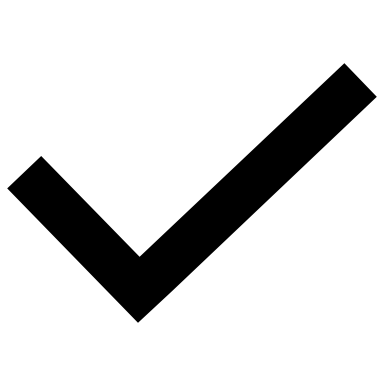 |  | 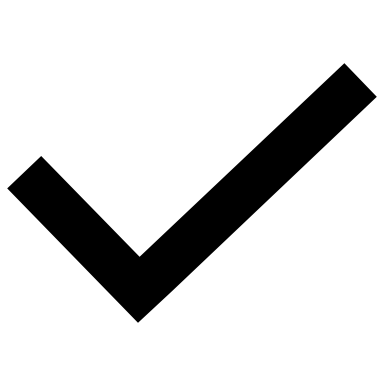 | Regional Cooperation | 2004-2017 |
| 6 | Economic Community of West African States | ECOWAS | 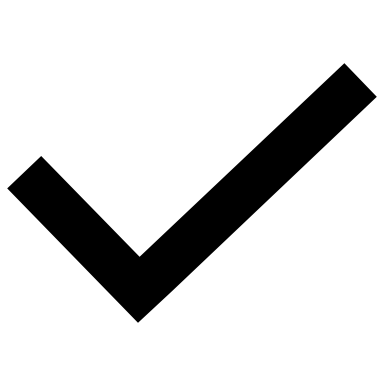 |  | 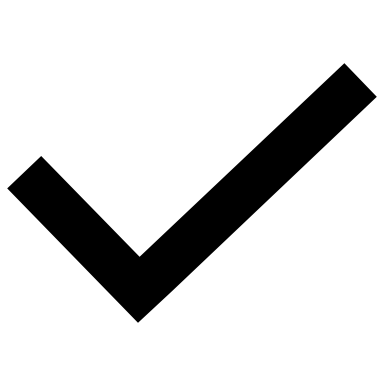 | Trade & Economy | 1991,1994, 1996, 1998-2017 |
| 7 | European Bank for Reconstruction and Development | EBRD |  | 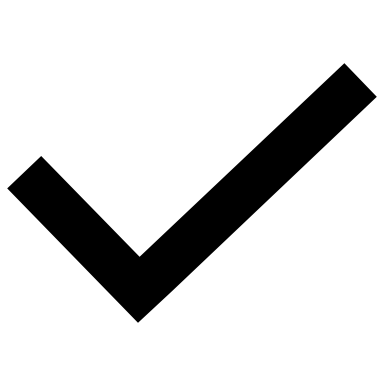 | 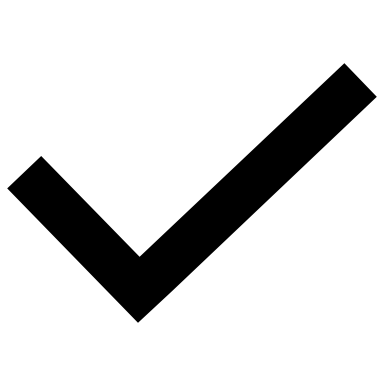 | Global Development Banking | 1997-2017 |
| 8 | European Union | EU | 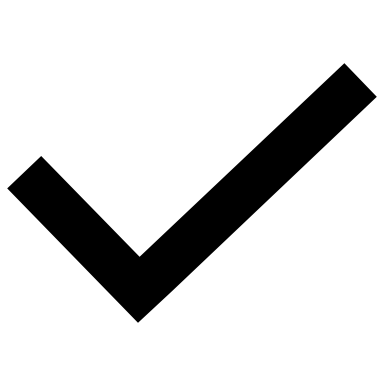 |  | 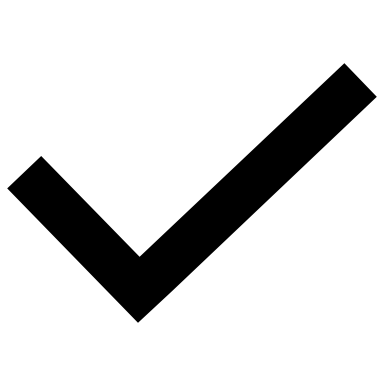 | Regional Cooperation | 1990-2017 |
| 9 | Food and Agriculture Organization | FAO |  | 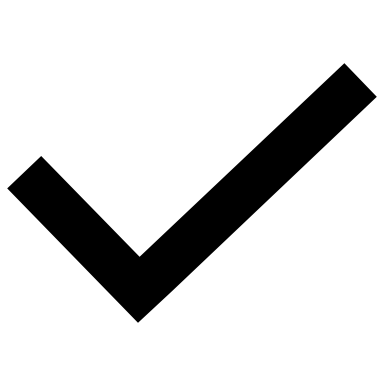 |  | Food & Agriculture | 1991-2017 |
| 10 | Inter-American Development Bank | IADB | 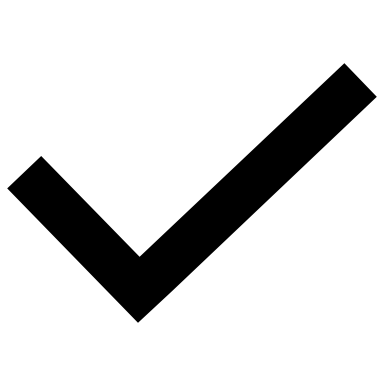 | 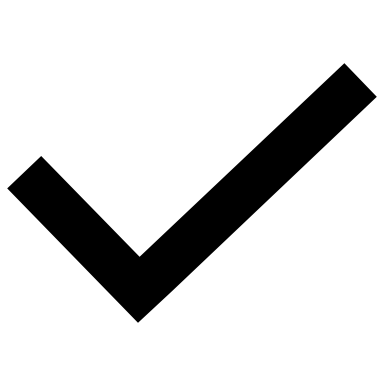 | 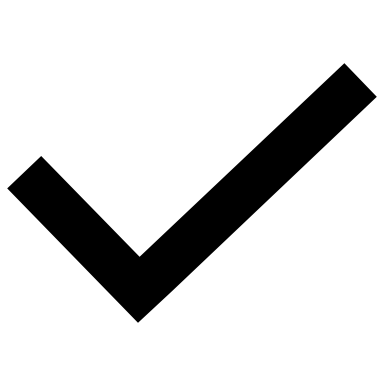 | Global Development Banking | 2000-2017 |
| 11 | International Fund for Agricultural Development | IFAD |  | 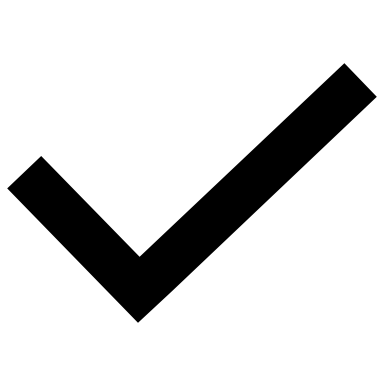 |  | Food & Agriculture | 2001-2017 |
| 12 | International Organization for Migration | IOM |  |  |  | Migration | 1999-2016 |
| 13 | North Atlantic Treaty Organization | NATO |  |  |  | Peace & Security | 2009-2016 |
| 14 | Organization of American States | OAS | 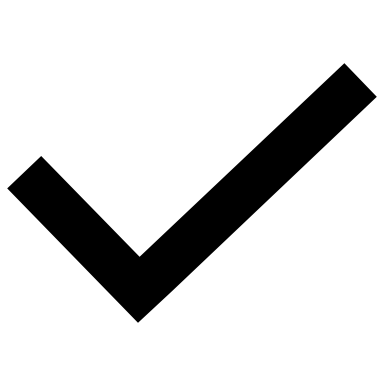 |  | 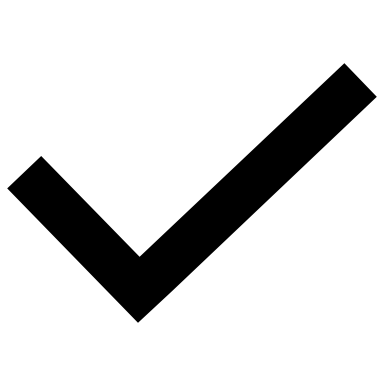 | Regional Cooperation | 2001-2017 |
| 15 | Organization for Economic Cooperation and Development | OECD |  |  |  | Development | 2000-2017 |
| 16 | Organization for Security and Defense and Cooperation in Europe | OSCE |  |  | 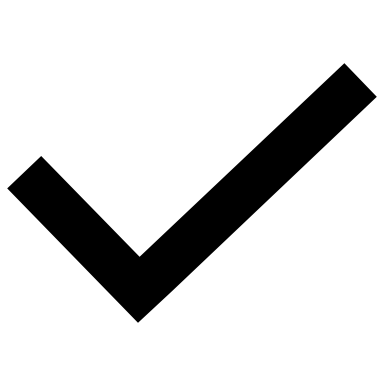 | Peace & Security | 2007-2017 |
| 17 | Pacific Islands Forum | PIF | 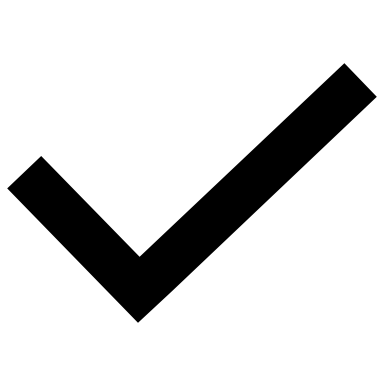 |  | 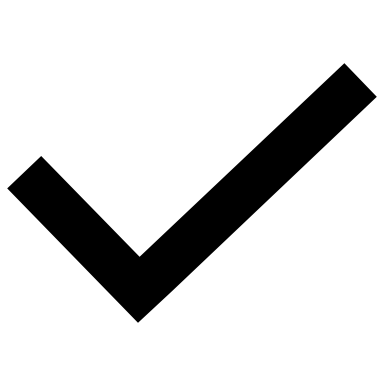 | Regional Cooperation | 2001-2016 |
| 18 | South Asian Association for Regional Cooperation | SAARC | 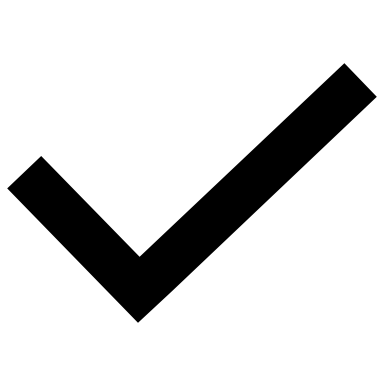 |  | 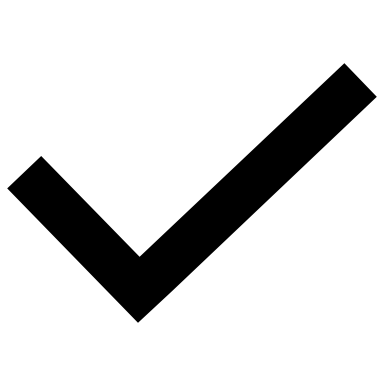 | Regional Cooperation | 1995-2016 |
| 19 | Southern African Development Community | SADC | 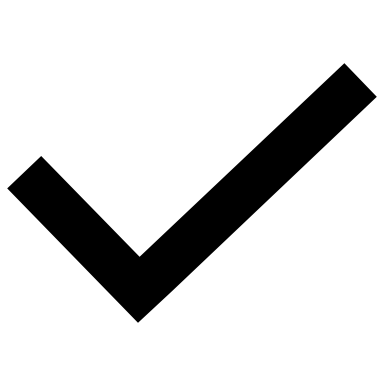 |  | 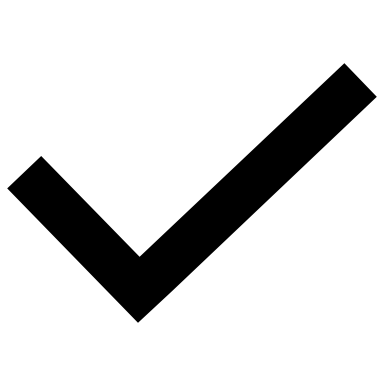 | Development | 1997-2015 |
| 20 | United Nations Development Programme | UNDP |  | 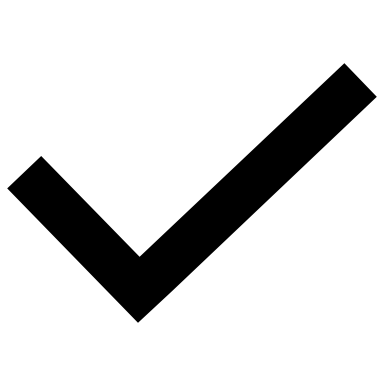 |  | Development | 2001-2017 |
| 21 | United Nations Population Fund | UNFPA |  |  |  | Health | 1997-2017 |
| 22 | United Nations Children's Emergency Fund | UNICEF |  |  |  | Development | 2009-2016 |
| 23 | UN High Commissioner for Refugees | UNHCR |  |  |  | Migration | 2007-2017 |
| 24 | United Nations Office for Disaster Risk Reduction | UNISDR |  |  |  | Disaster Risk Management | 2007-2017 |
| 25 | United Nations Office for the Coordination of Humanitarian Affairs | UNOCHA |  |  |  | Disaster Risk Management | 2005-2017 |
| 26 | United Nations Security Council | UNSC |  |  |  | Peace & Security | 1994-2017 |
| 27 | West Africa Rice Development Association | WARDA |  |  | 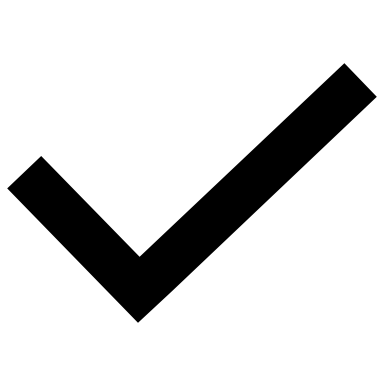 | Food & Agriculture | 2000-2016 |
| 28 | World Bank Group | WB/IBRD |  | 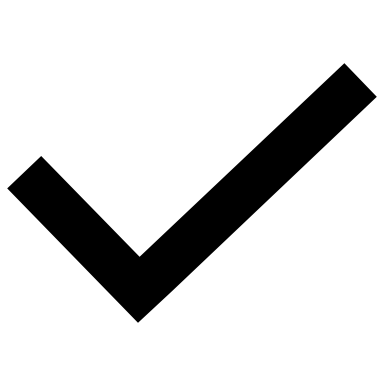 |  | Global Development Banking | 1990-2017 |
| 29 | World Health Organization | WHO |  |  |  | Health | 2008-2017 |
| 30 | World Trade Organization | WTO |  |  |  | Trade & Economy | 1991-2017 |

**Table S4.** Categorizing Types of Climate Adaptation Framings

| **Framing Type** | **Definition** |
| --- | --- |
| No mention | - |
| Economic framing | Climate risks for economic stability |
| Human security framing | Climate risks for human security |
| State security framing | Climate risks for state or national security |
| Energy security framing | Climate risks for energy security |

**Figure S1.** IO Annual Revenue and Adaptation Engagement Index

*Note*: Data averaged across the time span 1990-2017. Left-hand graph uses data from all 30 IOs, whereas right-hand graph excludes the EU. Source: UNSCEB. UN System Chief Executives Board for Coordination. Finance and Budget Data. Finance and Budget Data. 2021.Available from: https://unsceb.org/data-download.
